# Polycystic ovary syndrome (PCOS) and the risk of coronary heart disease (CHD): a meta-analysis

**DOI:** 10.18632/oncotarget.9553

**Published:** 2016-05-22

**Authors:** Luqian Zhao, Zhigang Zhu, Huiling Lou, Guodong Zhu, Weimin Huang, Shaogang Zhang, Feng Liu

**Affiliations:** ^1^ Department of Geriatrics, Guangzhou First People's Hospital, Guangzhou Medical University, Guangzhou, Guangdong, China

**Keywords:** PCOS, cardiovascular disease, association, meta-analysis, Pathology Section

## Abstract

Some studies reported a significant association between polycystic ovary syndrome (PCOS) and risk of cardiovascular disease (CVD). However, the results are controversial. A systematic search was conducted in the PubMed, Science Direct, EMBASE, and Cochrane Library databases. Five case-control studies and 5 cohort studies were selected, involving a total of 104392 subjects in this meta-analysis. PCOS was significantly associated with the increased risk of CVD (OR = 1.30; 95% CI 1.09 – 1.56; *P* = 0.004). In the subgroup analysis of study design, both case-control studies and prospective cohort studies showed significant results (OR = 1.79; 95% CI 1.16 – 2.77; *P* = 0.009; OR = 1.20; 95% CI 1.06 – 1.37; *P* = 0.005), while retrospective cohort studies did not show positive result (OR = 0.91; 95% CI 0.60 – 1.40; *P* = 0.68). In a further stratified analysis by type of CVD, a significant association was found between PCOS and coronary heart disease (CHD) (OR = 1.44; 95% CI 1.13 – 1.84; *P* = 0.004). However, no significant association was observed between PCOS and myocardial infarction (MI) (OR = 1.01; 95% CI 0.68 – 1.51; *P* = 0.95). In conclusion, this meta-analysis suggested that PCOS is significantly associated with increased CHD risk.

## INTRODUCTION

Polycystic ovary syndrome (PCOS) is one of the most common cause of infertility in women population [1]. Around 4% of unselected population of reproductive age and 7% of the caucasian population is believed to have this syndrome [2]. It is reported that PCOS patients had increased risk of many diseases, such as diabetes and infertility [3].

Cardiovascular disease (CVD) is one of the most common cause of mortality worldwide. Previous studies suggested that there will be more than 23 million CVD patients by 2030 [4-6]. The association between PCOS and risk of CVD has been reported by some studies. However, the results are controversial [7-16]. We thus did a meta-analysis and summarized the evidence on the incidence of CVD that has been studied in its association with PCOS.

## RESULTS

### Characteristics of eligible studies

**Figure 1:**
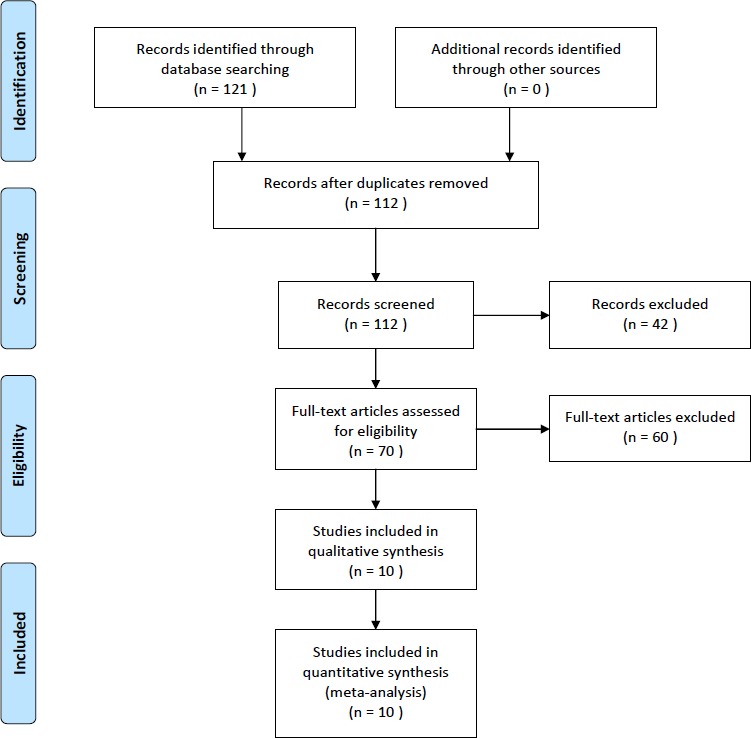
The selection of included studies

A total of 121 potential studies were found by searching online databases, such as PubMed, Web of Science, Science Direct, EMBASE, and Cochrane Library. Five case-control studies and five cohort studies were selected in this study. A total of 104392 subjects were included in this meta-analysis. The detailed literature search strategy was showed in Figure 1. The baseline characteristics, such as author name, publication year, ethnicity, design, age, outcomes, sample size, and covariants were depicted in Table 1. The quality of the studies was acceptable.

**Table 1:** Characteristics of the included studies

		Study			Follow-up	Sample			Newcastle-Ottawa
First author	Year	design	Race	Age	years	size	Outcomes	Covariants	Scale
Birdsall [7]	1997	CC	Caucasian	52.3	NA	142	MI	NA	7
Cibula [8]	2000	CC	Caucasian	52	NA	780	CHD	NA	5
Wild [9]	2000	CC	Caucasian	56.7	NA	1379	CHD	BMI	4
Solomon [10]	2002	PC	Caucasian	20-35	14	82439	CHD	Age, BMI, cigarette smoking, menopausal status/postmenopausal hormone use, parential history of MI before age 60 yr, parity, alcohol intake, aspirin use, multivitamin use, vitamin E supplement use, physical activity level, and history of oral contraceptive use	6
Krentz [11]	2007	CC	Caucasian	73.8	NA	713	CVD, CHD	NA	7
Lunde [12]	2007	CC	Caucasian	49.8	NA	854	MI	NA	4
Schmidt [13]	2011	PC	Caucasian	70.4	21	127	CVD, MI	NA	7
Wang [14]	2011	PC	Mixed	26	40	15005	CVD, CHD	Age, race, BMI, parity, current tobacco use, and oral contraceptive use	8
Iftikhar [15]	2012	RC	Caucasian	46.7	21	652	CVD, MI	Age at last follow-up, BMI, infertility treatment, postmenopausal hormone therapy, and family history of hypertension	7
Mani [16]	2013	RC	Caucasian	36	7	2301	MI	Age, BMI, Index of Multiple Deprivation as a marker of socio-economic status, hyperandrogenism, anovulation, ethnicity, smoking and history of hypertension	7

CC, case-control study; PC, prospective cohort study; RC, retrospective cohort study; CVD, cardiovascular disease; CHD, coronary heart disease; MI, myocardial infarction; BMI, body mass index; NA, not available.

### Association of PCOS and risk of CVD

**Figure 2:**
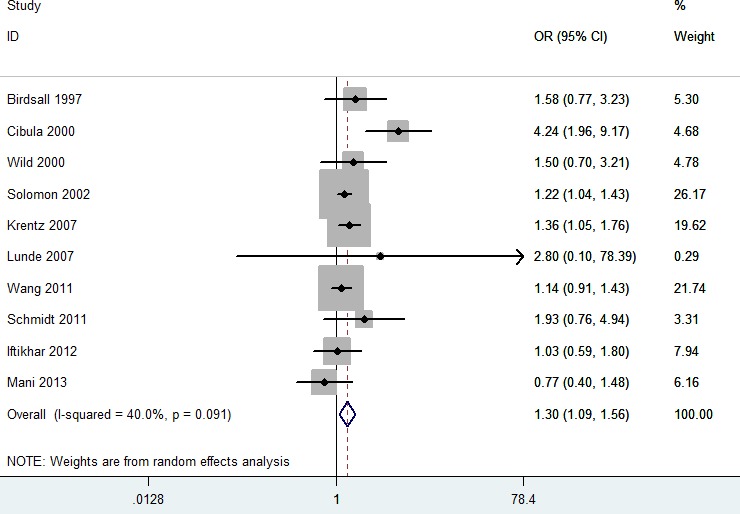
Meta-analysis of the association between PCOS and CVD

As shown in Figure 2, PCOS patients had significantly increased risk of CVD (OR = 1.30; 95% CI 1.09 - 1.56; *P* = 0.004). In the subgroup analysis of study design, both case-control studies and prospective cohort studies showed significant results (OR = 1.79; 95% CI 1.16 - 2.77; *P* = 0.009; OR = 1.20; 95% CI 1.06 - 1.37; *P* = 0.005), while retrospective cohort studies did not show positive result (OR = 0.91; 95% CI 0.60 - 1.40; *P* = 0.68). In a further subgroup analysis by the type of CVD, PCOS patients had significantly increased risk of coronary heart disease (CHD) (OR = 1.44; 95% CI 1.13 - 1.84; *P* = 0.004). However, PCOS patients did not have significantly increased risk of myocardial infarction (MI) (OR = 1.01; 95% CI 0.68 - 1.51; *P* = 0.95). In addition, both large sample size studies and small sample size studies showed significant results (OR = 1.18; 95% CI 1.04 - 1.34; *P* = 0.01; OR = 1.64; 95% CI 1.12 - 2.41; *P* = 0.01). The results were showed in Table 2.

**Table 2:** Results of this meta-analysis

	No. of study	OR (95% CI)	*P* Value	*I*^2^ (%)	*P* Value
CVD	10	1.30 (1.09-1.56)	0.004	40	0.09
Study design					
CC	5	1.79 (1.16-2.77)	0.009	48	0.11
PC	3	1.20 (1.06-1.37)	0.005	0	0.54
RC	2	0.91 (0.60-1.40)	0.68	0	0.50
Type of CVD					
CHD	5	1.44 (1.13-1.84)	0.004	59	0.04
MI	5	1.01 (0.68-1.51)	0.95	0	0.53
Sample size					
More than 1000 subjects	4	1.18 (1.04-1.34)	0.01	0	0.52
Less than 1000 subjects	6	1.64 (1.12-2.41)	0.01	48	0.09

CC, case-control study; PC, prospective cohort study; RC, retrospective cohort study; CVD, cardiovascular disease; CHD, coronary heart disease; MI, myocardial infarction

**Figure 3:**
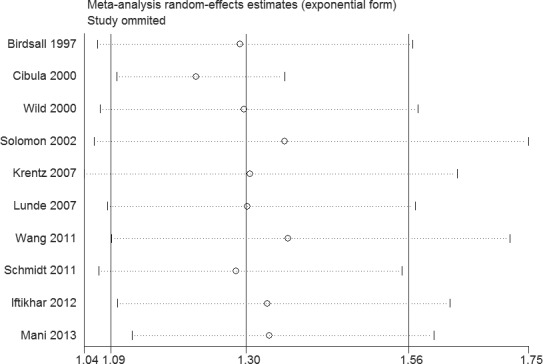
Sensitivity analysis of the association between PCOS and CVD

**Figure 4:**
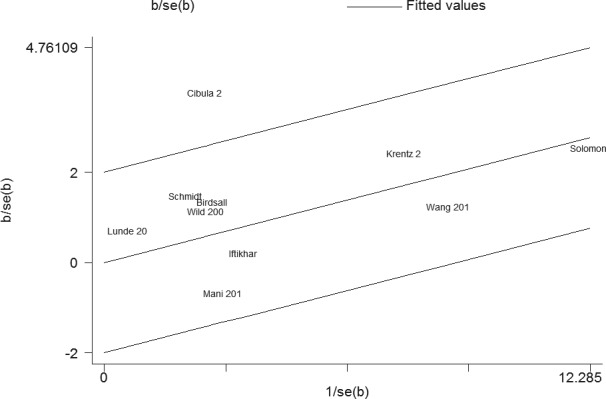
Galbraith plot of the association between PCOS and CVD

**Figure 5:**
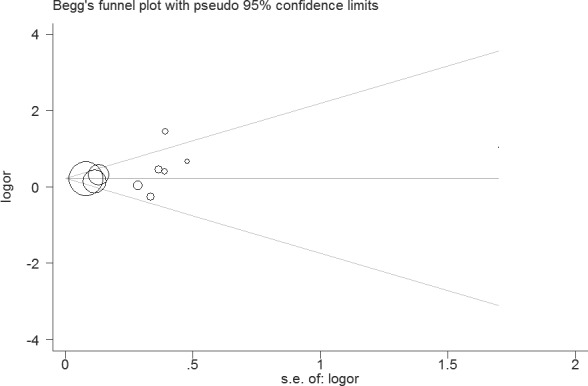
Funnel plot of the association between PCOS and CVD

Sensitivity analysis suggested that the results of this study were statistically reliable (Figure 3). As shown in Figure 4, one study was the outlier [8]. After excluding this study, no heterogeneity was found (I^2^ = 0%, *P* = 0.74). Furthermore, the result was not influenced (OR = 1.22, 95% CI 1.10 - 1.36, *P* = 0.0003). In addition, Begg's funnel plot (Figure 5) and Egger's test indicated no significant publication bias (*P* = 0.261).

## DISCUSSION

This is a meta-analysis which aimed to clarify the association between PCOS and the risk of CVD. Ten studies involving more than 100000 individuals were included in our meta-analysis. The results suggested that PCOS was a risk factor of the increased risk of CVD. In a stratified analysis by type of CVD, a significant association was found between PCOS and CHD. However, no significant association was observed between PCOS and MI. Two previous meta-analyses also found that women with PCOS appeared to be at increased risk of CHD [17, 18]. Thus, this present study had three advantages. First, more studies were included in this meta-analysis. Second, we investigated the association between PCOS and MI risk for the first time. Third, sensitivity analysis and Galbraith plot were investigated in this meta-analysis.

A recent meta-analysis determined the relationship between PCOS and CVD risk markers [19]. That study suggested that PCOS patients had significantly elevated homocysteine, dimethylarginine, and lipoprotein, et al. [19]. In addition, Ozegowska et al. indicated that PCOS patients had higher CVD risk factors, such as body mass index (BMI), waist circumference, and waist-to-hip ratio [20]. Therefore, patients with PCOS might have high risk of CVD.

This study had some advantages. First, substantial number of individuals were pooled from different trials, which significantly increased statistical power of the meta-analysis. Second, the quality of studies included in current meta-analysis was relatively satisfactory and met our predefined inclusion criteria. Third, no publication bias was found, which suggested that the result was unbiased.

Several limitations of this study must be acknowledged. Firstly, only online databases were searched; some studies may have been missed. Secondly, no studies with Asians and other races was included in this meta-analysis. Thirdly, heterogeneity was found in this study. Sensitivity analysis was used to find the source of heterogeneity. Fortunately, it was decreased when one outlier was excluded.

This study suggested that PCOS patients had significantly increased CHD risk.

## MATERIALS AND METHODS

### Publication search

PubMed, Science Direct, EMBASE, and Cochrane Library databases were searched by two independent authors (LQ Zhao and ZG Zhu). Last search was updated in Apr, 2016. The search terms were used as follows: (polycystic ovary syndrome or PCOS) and (cardiovascular diseases or CVD). No publication date or language restrictions were imposed. We contacted primary authors to clear doubts. The reference list of obtained articles is showed in the supplemental material.

### Study selection

The selection criteria of the retrieved articles in our meta-analysis were as follows: a) case-control studies or cohort studies; b) studies evaluating the association between PCOS and risk of CVD; c) sufficient data available to calculate an odds ratio (OR) with 95% confidence interval (CI). The exclusion criteria of the meta-analysis were: a) case-only studies; b) studies with incomplete data; and c) meta-analyses, letters, reviews, and editorial articles. If more than one study was published by the same author using the same patient population or overlapping case series, the study with the largest size of samples was included.

### Data extraction and qualitative assessment

Two investigators extracted data from the included studies independently, and the respective studies were retrieved for further consideration if judged pertinent by one or two reviewers. Any discrepancies were identified and resolved by consensus. For each study, the following data were extracted: first author's name, year of publication, study design, race, age, duration of follow-up, sample size, outcomes, and covariant. A modification of the Newcastle-Ottawa Scale (NOS) was used as an assessment tool for selection, comparability, and outcome assessment. The studies should (1) brief description of appropriateness of studies assembled for assessing the hypothesis; (2) rationale for the selection and coding of data (eg. Roterdam PCOS Consensus, NIH criteria); (3) documentation of how data were classified (eg. multiple raters blinding, interrater reliability); (4) assessment of confounding variables (eg, comparability of cases and controls in studies where appropriate); (5) assessment of heterogeneity.

### Statistical analysis

The strength of the association between PCOS and CVD risk was estimated by calculating ORs with 95% CIs. Tests for heterogeneity were made among studies using the Cochran's Q and I^2^ test statistic. For the Cochran's Q test statistic, a *P* value < 0.10 was accepted as statistically significant heterogeneity. Random-effects models were used to estimate summary ORs and 95% CIs. We also conducted subgroup analyses by study design, type of outcomes, and sample size. Galbraith plot was also performed to identify sources of heterogeneity. Sensitivity analyses were conducted to assess the strength of our findings by excluding one study at a time. Begg's funnel plot and Egger's regression test were used to evaluate publication bias. In Egger's test, when *P* value < 0.10, it was considered statistically significant publication bias. All analyses were conducted using Stata v.12 (StataCorp LP, TX) statistical software.

## SUPPLEMENTARY MATERIALS


