# Supplementary material for: Polycystic ovary syndrome (PCOS) and the risk of coronary heart disease (CHD): a meta-analysis
Source: Oncotarget. 2016 May 22;7(23):33715–21. doi: 10.18632/oncotarget.9553 (PMC5085114; doi:10.18632/oncotarget.9553)
Supplement: Supplementary file 1 [file oncotarget-07-33715-s001.pdf]

Polycystic ovary syndrome (PCOS) and the risk of coronary heart disease (CHD): a meta-analysis

Supplementary Material

1: El Hayek S, Bitar L, Hamdar LH, Mirza FG, Daoud G. Poly Cystic Ovarian Syndrome: An Updated Overview. Front Physiol. 2016 Apr 5;7:124. doi: 10.3389/fphys.2016.00124. eCollection 2016.

2: Macut D, Tziomalos K, Božić-Antić I, Bjekić-Macut J, Katsikis I, Papadakis E, Andrić Z, Panidis D. Non-alcoholic fatty liver disease is associated with insulin resistance and lipid accumulation product in women with polycystic ovary syndrome. Hum Reprod. 2016 Apr 12. pii: dew076. [Epub ahead of print]

3: Ouyang P, Wenger NK, Taylor D, Rich-Edwards JW, Steiner M, Shaw LJ, Berga SL, Miller VM, Merz NB. Strategies and methods to study female-specific cardiovascular health and disease: a guide for clinical scientists. Biol Sex Differ. 2016 Mar 31;7:19. doi: 10.1186/s13293-016-0073-y. eCollection 2016.

4: Kim JY, Tfayli H, Michaliszyn SF, Lee S, Arslanian S. Distinguishing characteristics of metabolically healthy versus metabolically unhealthy obese adolescent girls with polycystic ovary syndrome. Fertil Steril. 2016 Feb 24. pii: S0015-0282(16)00088-1. doi: 10.1016/j.fertnstert.2016.02.004. [Epub ahead of print]

5: Vipin VP, Dabadghao P, Shukla M, Kapoor A, Raghuvanshi AS, Ramesh V. Cardiovascular disease risk in first-degree relatives of women with polycystic ovary syndrome. Fertil Steril. 2016 Feb 4. pii: S0015-0282(16)00065-0. doi: 10.1016/j.fertnstert.2016.01.024. [Epub ahead of print]

6: Ozegowska K, Pawelczyk L. Cardiometabolic risk in patients with polycystic ovary syndrome. Ginekol Pol. 2015 Nov;86(11):840-8.

7: Ozler S, Oztas E, Tokmak A, Ergin M, Isci E, Eren F, Pehlivan S, Neselioglu S, Yılmaz N. The association of thiol/disulphide homeostasis and lipid accumulation index with cardiovascular risk factors in overweight adolescents with polycystic ovary syndrome. Clin Endocrinol (Oxf). 2016 Apr;84(4):516-23. doi: 10.1111/cen.12965. Epub 2015 Nov 25.

8: Jędrzejuk D, Lwow F, Kuliczkowska-Płaksej J, Hirnle L, Trzmiel-Bira A, Lenarcik-Kabza A, Kolackov K, Łaczmański Ł, Milewicz A. Association of serum glypican-4 levels with cardiovascular risk predictors in women with polycystic

ovary syndrome - a pilot study. Gynecol Endocrinol. 2016 Mar;32(3):223-6. doi: 10.3109/09513590.2015.1110137. Epub 2015 Nov 16.

9: Anastasia K, Koika V, Roupas ND, Armeni A, Marioli D, Panidis D, George A, Georgopoulos NA. Association of Calpain (CAPN) 10 (UCSNP-43, rs3792267) gene polymorphism with elevated serum androgens in young women with the most severe phenotype of polycystic ovary syndrome (PCOS). Gynecol Endocrinol. 2015;31(8):630-4. doi: 10.3109/09513590.2015.1032932. Epub 2015 Sep 17.

10: Enkhmaa B, Anuurad E, Zhang W, Abbuthalha A, Kaur P, Visla J, Karakas S, Berglund L. Lipoprotein(a) and apolipoprotein(a) in polycystic ovary syndrome. Clin Endocrinol (Oxf). 2015 Sep 4. doi: 10.1111/cen.12937. [Epub ahead of print]

11: Aziz M, Sidelmann JJ, Wissing ML, Faber J, Skouby SO. Endogenous thrombin potential in polycystic ovary syndrome: the association to body mass index, insulin resistance, and inflammation. Gynecol Endocrinol. 2015;31(9):720-4. doi: 10.3109/09513590.2015.1032930. Epub 2015 Aug 17.

12: Desai NA, Patel SS. Increased insulin-like growth factor-1 in relation to cardiovascular function in polycystic ovary syndrome: friend or foe? Gynecol Endocrinol. 2015 Oct;31(10):801-7. doi: 10.3109/09513590.2015.1075497. Epub 2015 Aug 18.

13: Daan NM, Jaspers L, Koster MP, Broekmans FJ, de Rijke YB, Franco OH, Laven JS, Kavousi M, Fauser BC. Androgen levels in women with various forms of ovarian dysfunction: associations with cardiometabolic features. Hum Reprod. 2015 Oct;30(10):2376-86. doi: 10.1093/humrep/dev195. Epub 2015 Aug 12.

14: Ali AT. Polycystic ovary syndrome and metabolic syndrome. Ceska Gynekol. 2015 Aug;80(4):279-89. Review.

15: Glintborg D, Sidelmann JJ, Altinok ML, Mumm H, Andersen M. Increased thrombin generation in women with polycystic ovary syndrome: A pilot study on the effect of metformin and oral contraceptives. Metabolism. 2015 Oct;64(10):1272-8. doi: 10.1016/j.metabol.2015.06.011. Epub 2015 Jun 17.

16: Cobin RH. COMMENTARY ON THE ROLE OF INSULIN SENSITIZERS ON CARDIOVASCULAR RISK FACTORS IN POLYCYSTIC OVARIAN SYNDROME: A META-ANALYSIS. Endocr Pract. 2015 Jun;21(6):700-3. doi: 10.4158/EP15713.CO.

17: Nascimento JX, Chein MB, de Sousa RM, Ferreira Ados S, Navarro PA, Brito LM.

Importance of lipid accumulation product index as a marker of CVD risk in PCOS women. Lipids Health Dis. 2015 Jun 24;14:62. doi: 10.1186/s12944-015-0061-y.

18: Baer TE, Milliren CE, Walls C, DiVasta AD. Clinical Variability in Cardiovascular Disease Risk Factor Screening and Management in Adolescent and Young Adult Women with Polycystic Ovary Syndrome. J Pediatr Adolesc Gynecol. 2015 Oct;28(5):317-23. doi: 10.1016/j.jpag.2014.09.010. Epub 2014 Oct 8.

19: Hyderali BN, Mala K. Oxidative stress and cardiovascular complications in polycystic ovarian syndrome. Eur J Obstet Gynecol Reprod Biol. 2015 Aug;191:15-22. doi: 10.1016/j.ejogrb.2015.05.005. Epub 2015 Jun 2.

20: Hoang V, Bi J, Mohankumar SM, Vyas AK. Liraglutide improves hypertension and metabolic perturbation in a rat model of polycystic ovarian syndrome. PLoS One. 2015 May 26;10(5):e0126119. doi: 10.1371/journal.pone.0126119. eCollection 2015.

21: Lam DW, LeRoith D. Metabolic Syndrome. 2015 May 19. In: De Groot LJ, Beck-Peccoz P, Chrousos G, Dungan K, Grossman A, Hershman JM, Koch C, McLachlan R, New M, Rebar R, Singer F, Vinik A, Weickert MO, editors. Endotext [Internet]. South Dartmouth (MA): MDText.com, Inc.; 2000-. Available from http://www.ncbi.nlm.nih.gov/books/NBK278936/

22: Macut D, Bačević M, Božić-Antić I, Bjekić-Macut J, Čivčić M, Erceg S, Vojnović Milutinović D, Stanojlović O, Andrić Z, Kastratović-Kotlica B, Šukilović T. Predictors of subclinical cardiovascular disease in women with polycystic ovary syndrome: interrelationship of dyslipidemia and arterial blood pressure. Int J Endocrinol. 2015;2015:812610. doi: 10.1155/2015/812610. Epub 2015 Mar 24.

23: Cahill DJ, O'Brien K. Polycystic ovary syndrome (PCOS): metformin. BMJ Clin Evid. 2015 Mar 27;2015. pii: 1408.

24: Thethi TK, Katalenich B, Nagireddy P, Chabbra P, Md NK, Fonseca V. ROLE OF INSULIN SENSITIZERS ON CARDIOVASCULAR RISK FACTORS IN POLYCYSTIC OVARIAN SYNDROME: A META-ANALYSIS. Endocr Pract. 2015 Jun;21(6):645-67. doi: 10.4158/EP14396.RA. Epub 2015 Feb 25.

25: Mousa A, Naderpoor N, Teede HJ, De Courten MP, Scragg R, De Courten B. Vitamin D and cardiometabolic risk factors and diseases. Minerva Endocrinol. 2015

Sep;40(3):213-30. Epub 2015 Feb 25.

26: Appelman Y, van Rijn BB, Ten Haaf ME, Boersma E, Peters SA. Sex differences in cardiovascular risk factors and disease prevention. Atherosclerosis. 2015 Jul;241(1):211-8. doi: 10.1016/j.atherosclerosis.2015.01.027. Epub 2015 Jan 28.

27: Daskalopoulos G, Karkanaki A, Piouka A, Prapas N, Panidis D, Gkeleris P, Athyros VG. Excess Metabolic and Cardiovascular Risk is not Manifested in all Phenotypes of Polycystic Ovary Syndrome: Implications for Diagnosis and Treatment. Curr Vasc Pharmacol. 2015;13(6):788-800.

28: Macut D, Antić IB, Bjekić-Macut J. Cardiovascular risk factors and events in women with androgen excess. J Endocrinol Invest. 2015 Mar;38(3):295-301. doi: 10.1007/s40618-014-0215-1. Epub 2014 Nov 29.

29: Calderon-Margalit R, Siscovick D, Merkin SS, Wang E, Daviglus ML, Schreiner PJ, Sternfeld B, Williams OD, Lewis CE, Azziz R, Schwartz SM, Wellons MF. Prospective association of polycystic ovary syndrome with coronary artery calcification and carotid-intima-media thickness: the Coronary Artery Risk Development in Young Adults Women's study. Arterioscler Thromb Vasc Biol. 2014 Dec;34(12):2688-94. doi: 10.1161/ATVBAHA.114.304136. Epub 2014 Oct 30.

30: Desai V, Prasad NR, Manohar SM, Sachan A, Narasimha SR, Bitla AR. Oxidative stress in non-obese women with polycystic ovarian syndrome. J Clin Diagn Res. 2014 Jul;8(7):CC01-3. doi: 10.7860/JCDR/2014/8125.4530. Epub 2014 Jul 20.

31: Karbek B, Ozbek M, Karakose M, Topaloglu O, Bozkurt NC, Cakır E, Aslan MS, Delibasi T. Copeptin, a surrogate marker for arginine vasopressin, is associated with cardiovascular risk in patients with polycystic ovary syndrome. J Ovarian Res. 2014 Mar 14;7:31. doi: 10.1186/1757-2215-7-31.

32: Snyder ML, Shields KJ, Korytkowski MT, Sutton-Tyrrell K, Talbott EO. Complement protein C3 and coronary artery calcium in middle-aged women with polycystic ovary syndrome and controls. Gynecol Endocrinol. 2014 Jul;30(7):511-5. doi: 10.3109/09513590.2014.895985. Epub 2014 Mar 5.

33: Dardzińska JA, Rachoń D, Kuligowska-Jakubowska M, Aleksandrowicz-Wrona E, Płoszyński A, Wyrzykowski B, Lysiak-Szydłowska W. Effects of metformin or an oral contraceptive containing cyproterone acetate on serum c-reactive protein,

interleukin-6 and soluble vascular cell adhesion molecule-1 concentrations in women with polycystic ovary syndrome. Exp Clin Endocrinol Diabetes. 2014 Feb;122(2):118-25. doi: 10.1055/s-0033-1363261. Epub 2014 Feb 19.

34: Sahin SB, Cure MC, Ugurlu Y, Ergul E, Gur EU, Alyildiz N, Bostan M. Epicardial adipose tissue thickness and NGAL levels in women with polycystic ovary syndrome. J Ovarian Res. 2014 Feb 16;7:24. doi: 10.1186/1757-2215-7-24.

35: Pourteymour Fard Tabrizi F, Alipoor B, Mehrzad Sadaghiani M, Ostadrahimi A, Malek Mahdavi A. Metabolic Syndrome and Its Characteristics among Reproductive-Aged Women with Polycystic Ovary Syndrome: A Cross-sectional Study in Northwest Iran. Int J Fertil Steril. 2013 Jan;6(4):244-9. Epub 2013 Mar 3.

36: Roe A, Hillman J, Butts S, Smith M, Rader D, Playford M, Mehta NN, Dokras A. Decreased cholesterol efflux capacity and atherogenic lipid profile in young women with PCOS. J Clin Endocrinol Metab. 2014 May;99(5):E841-7. doi: 10.1210/jc.2013-3918. Epub 2014 Feb 10.

37: Aziz M, Wissing ML, Naver KV, Faber J, Skouby SO. Polycystic ovary syndrome and low-grade inflammation with special reference to YKL-40. Gynecol Endocrinol. 2014 Apr;30(4):311-5. doi: 10.3109/09513590.2013.879854. Epub 2014 Jan 28.

38: Mani H, Levy MJ, Davies MJ, Morris DH, Gray LJ, Bankart J, Blackledge H, Khunti K, Howlett TA. Diabetes and cardiovascular events in women with polycystic ovary syndrome: a 20-year retrospective cohort study. Clin Endocrinol (Oxf). 2013 Jun;78(6):926-34. doi: 10.1111/cen.12068. Epub 2013 Apr 6.

39: Hillman JK, Johnson LN, Limaye M, Feldman RA, Sammel M, Dokras A. Black women with polycystic ovary syndrome (PCOS) have increased risk for metabolic syndrome and cardiovascular disease compared with white women with PCOS [corrected]. Fertil Steril. 2014 Feb;101(2):530-5. doi: 10.1016/j.fertnstert.2013.10.055. Epub 2013 Dec 30. Erratum in: Fertil Steril. 2014 Jun;101(6):1791.

40: Kim JJ, Choi YM, Kang JH, Hwang KR, Chae SJ, Kim SM, Ku SY, Kim SH, Kim JG, Moon SY. Carotid intima-media thickness in mainly non-obese women with polycystic ovary syndrome and age-matched controls. Obstet Gynecol Sci. 2013 Jul;56(4):249-55. doi: 10.5468/ogs.2013.56.4.249. Epub 2013 Jul 15.

41: Sprung VS, Cuthbertson DJ, Pugh CJ, Aziz N, Kemp GJ, Daousi C, Green DJ, Cable NT, Jones H. Exercise training in polycystic ovarian syndrome enhances flow-mediated dilation in the absence of changes in fatness. Med Sci Sports Exerc. 2013 Dec;45(12):2234-42. doi: 10.1249/MSS.0b013e31829ba9a1. P

42: Armeni E, Stamatelopoulos K, Rizos D, Georgiopoulos G, Kazani M, Kazani A, Kolyviras A, Stellos K, Panoulis K, Alexandrou A, Creatsa M, Papamichael C, Lambrinoudaki I. Arterial stiffness is increased in asymptomatic nondiabetic postmenopausal women with a polycystic ovary syndrome phenotype. J Hypertens. 2013 Oct;31(10):1998-2004. doi: 10.1097/HJH.0b013e3283630362. Erratum in: J Hypertens. 2014 Mar;32(3):702.

43: Khalil RA. Estrogen, vascular estrogen receptor and hormone therapy in postmenopausal vascular disease. Biochem Pharmacol. 2013 Dec 15;86(12):1627-42. doi: 10.1016/j.bcp.2013.09.024. Epub 2013 Oct 4.

44: Guleria AK, Syal SK, Kapoor A, Kumar S, Tiwari P, Dabadghao P. Cardiovascular disease risk in young Indian women with polycystic ovary syndrome. Gynecol Endocrinol. 2014 Jan;30(1):26-9. doi: 10.3109/09513590.2013.831835.

45: Rochester JR. Bisphenol A and human health: a review of the literature. Reprod Toxicol. 2013 Dec;42:132-55. doi: 10.1016/j.reprotox.2013.08.008. Epub 2013 Aug 30.

46: Sprung VS, Jones H, Pugh CJ, Aziz NF, Daousi C, Kemp GJ, Green DJ, Cable NT, Cuthbertson DJ. Endothelial dysfunction in hyperandrogenic polycystic ovary syndrome is not explained by either obesity or ectopic fat deposition. Clin Sci (Lond). 2014 Jan 1;126(1):67-74. doi: 10.1042/CS20130186.

47: Schenck-Gustafsson K, Rees M. Cardiology for gynecologists--a minireview. Maturitas. 2013 Aug;75(4):386-91. doi: 10.1016/j.maturitas.2013.04.018. Epub 2013 May 31.

48: Gözdemir E, Kaygusuz I, Kafalı H. Is hepcidin a new cardiovascular risk marker in polycystic ovary syndrome? Gynecol Obstet Invest. 2013;75(3):196-202. doi: 10.1159/000348497. Epub 2013 Mar 15.

49: Thomson RL, Spedding S, Brinkworth GD, Noakes M, Buckley JD. Seasonal effects on vitamin D status influence outcomes of lifestyle intervention in overweight and obese women with polycystic ovary syndrome. Fertil Steril. 2013

May;99(6):1779-85. doi: 10.1016/j.fertnstert.2012.12.042. Epub 2013 Jan 26.

50: Cakir E, Ozbek M, Sahin M, Cakal E, Gungunes A, Ginis Z, Demirci T, Delibasi T. Heart type fatty acid binding protein response and subsequent development of atherosclerosis in insulin resistant polycystic ovary syndrome patients. J Ovarian Res. 2012 Dec 18;5(1):45. doi: 10.1186/1757-2215-5-45.

51: Huang G, Coviello A. Clinical update on screening, diagnosis and management of metabolic disorders and cardiovascular risk factors associated with polycystic ovary syndrome. Curr Opin Endocrinol Diabetes Obes. 2012 Dec;19(6):512-9. doi: 10.1097/MED.0b013e32835a000e.

52: Choi YS, Yang HI, Cho S, Jung JA, Jeon YE, Kim HY, Seo SK, Lee BS. Serum asymmetric dimethylarginine, apelin, and tumor necrosis factor-α levels in non-obese women with polycystic ovary syndrome. Steroids. 2012 Nov;77(13):1352-8. doi: 10.1016/j.steroids.2012.08.005. Epub 2012 Aug 25.

53: Hudecova M, Jan H, Christian B, Poromaa Inger S. Long-term reproductive and metabolic consequences of PCOS. Curr Diabetes Rev. 2012 Nov;8(6):444-51.

54: Sprung VS, Atkinson G, Cuthbertson DJ, Pugh CJ, Aziz N, Green DJ, Cable NT, Jones H. Endothelial function measured using flow-mediated dilation in polycystic ovary syndrome: a meta-analysis of the observational studies. Clin Endocrinol (Oxf). 2013 Mar;78(3):438-46. doi: 10.1111/j.1365-2265.2012.04490.x.

55: Mishra A, Younossi ZM. Epidemiology and Natural History of Non-alcoholic Fatty Liver Disease. J Clin Exp Hepatol. 2012 Jun;2(2):135-44. doi: 10.1016/S0973-6883(12)60102-9. Epub 2012 Jul 21.

56: Ganie MA, Farooqui KJ, Bhat MA, Mir MM, Shah ZA, Douhath S, Mir SH, Rashid F, Naqshi S, Masoodi MI, Zargar SA, Zargar AH. Pattern of urinary albumin excretion in normotensive young and adolescent Indian women with polycystic ovary syndrome. Indian J Endocrinol Metab. 2012 Mar;16(2):277-82. doi: 10.4103/2230-8210.93752.

57: Iftikhar S, Collazo-Clavell ML, Roger VL, St Sauver J, Brown RD Jr, Cha S, Rhodes DJ. Risk of cardiovascular events in patients with polycystic ovary syndrome. Neth J Med. 2012 Mar;70(2):74-80.

58: Sirmans SM, Weidman-Evans E, Everton V, Thompson D. Polycystic ovary syndrome and chronic inflammation: pharmacotherapeutic implications. Ann Pharmacother. 2012 Mar;46(3):403-18. doi: 10.1345/aph.1Q514. Epub 2012 Feb 28.

59: Wang ET, Cirillo PM, Vittinghoff E, Bibbins-Domingo K, Cohn BA, Cedars MI. Menstrual irregularity and cardiovascular mortality. J Clin Endocrinol Metab. 2011 Jan;96(1):E114-8. doi: 10.1210/jc.2010-1709. Epub 2010 Oct 27.

60: Meyer ML, Malek AM, Wild RA, Korytkowski MT, Talbott EO. Carotid artery intima-media thickness in polycystic ovary syndrome: a systematic review and meta-analysis. Hum Reprod Update. 2012 Mar-Apr;18(2):112-26. doi: 10.1093/humupd/dmr046. Epub 2011 Nov 22.

61: Schmidt J, Landin-Wilhelmsen K, Brännström M, Dahlgren E. Cardiovascular disease and risk factors in PCOS women of postmenopausal age: a 21-year controlled follow-up study. J Clin Endocrinol Metab. 2011 Dec;96(12):3794-803. doi: 10.1210/jc.2011-1677. Epub 2011 Sep 28.

62: Dessapt-Baradez C, Reza M, Sivakumar G, Hernandez-Fuentes M, Markakis K, Gnudi L, Karalliedde J. Circulating vascular progenitor cells and central arterial stiffness in polycystic ovary syndrome. PLoS One. 2011;6(5):e20317. doi: 10.1371/journal.pone.0020317. Epub 2011 May 31.

63: Toulis KA, Goulis DG, Mintziori G, Kintiraki E, Eukarpidis E, Mouratoglou SA, Pavlaki A, Stergianos S, Poulasouchidou M, Tzellos TG, Makedos A, Chourdakis M, Tarlatzis BC. Meta-analysis of cardiovascular disease risk markers in women with polycystic ovary syndrome. Hum Reprod Update. 2011 Nov-Dec;17(6):741-60. doi: 10.1093/humupd/dmr025.

64: Moran LJ, Cameron JD, Strauss BJ, Teede HJ. Vascular function in the diagnostic categories of polycystic ovary syndrome. Hum Reprod. 2011 Aug;26(8):2192-9. doi: 10.1093/humrep/der159. Epub 2011 May 25.

65: Taylor MC, Reema Kar A, Kunselman AR, Stetter CM, Dunaif A, Legro RS. Evidence for increased cardiovascular events in the fathers but not mothers of women with polycystic ovary syndrome. Hum Reprod. 2011 Aug;26(8):2226-31. doi: 10.1093/humrep/der101. Epub 2011 Apr 19.

66: Bruce KD, Cagampang FR. Epigenetic priming of the metabolic syndrome. Toxicol Mech Methods. 2011 May;21(4):353-61. doi: 10.3109/15376516.2011.559370.

67: Daskalopoulos GN, Karkanaki A, Karagiannis A, Mikhailidis DP, Athyros VG. Is the risk for cardiovascular disease increased in all phenotypes of the polycystic ovary syndrome? Angiology. 2011 May;62(4):285-90. doi: 10.1177/0003319711399571.

68: Burchall G, Linden MD, Teede H, Piva TJ. Hemostatic abnormalities and relationships to metabolic and hormonal status in polycystic ovarian syndrome. Trends Cardiovasc Med. 2011 Jan;21(1):6-14. doi: 10.1016/j.tcm.2012.01.001.

69: Cakir E, Ozbek M, Ozkaya E, Colak N, Cakal E, Sayki M, Gungunes A, Aliyazicioglu Y, Mentese A, Delibasi T. Oxidative stress markers are not valuable markers in lean and early age of polycystic ovary syndrome patients. J Endocrinol Invest. 2011 Jul-Aug;34(7):e178-82. doi: 10.3275/7352. Epub 2010 Nov 16.

70: Caglar GS, Oztas E, Karadag D, Pabuccu R, Eren AA. The association of urinary albumin excretion and metabolic complications in polycystic ovary syndrome. Eur J Obstet Gynecol Reprod Biol. 2011 Jan;154(1):57-61. doi: 10.1016/j.ejogrb.2010.08.024.

71: Moradi S, Mollabashi M, Kerman SR. Relation between C-reactive protein and body mass index in patients with polycystic ovarian syndrome. Gynecol Endocrinol. 2011 Jul;27(7):480-5. doi: 10.3109/09513590.2010.501876. Epub 2010 Sep 14.

72: Gingnell M, Dahlbom I, Lindholm A, Hudecova M, Arnadottir R, Hansson T, Sundstrom-Poromaa I. Patients with polycystic ovary syndrome have lower levels of IgM anti-phosphorylcholine antibodies than healthy women. Gynecol Endocrinol. 2011 Jul;27(7):486-90. doi: 10.3109/09513590.2010.501880. Epub 2010 Jul 21.

73: Veltman-Verhulst SM, van Rijn BB, Westerveld HE, Franx A, Bruinse HW, Fauser BC, Goverde AJ. Polycystic ovary syndrome and early-onset preeclampsia: reproductive manifestations of increased cardiovascular risk. Menopause. 2010 Sep-Oct;17(5):990-6. doi: 10.1097/gme.0b013e3181ddf705.

74: Tomlinson J, Millward A, Stenhouse E, Pinkney J. Type 2 diabetes and cardiovascular disease in polycystic ovary syndrome: what are the risks and can

they be reduced? Diabet Med. 2010 May;27(5):498-515. doi:
10.1111/j.1464-5491.2010.02994.x.

75: Wild RA, Carmina E, Diamanti-Kandarakis E, Dokras A, Escobar-Morreale HF, Futterweit W, Lobo R, Norman RJ, Talbott E, Dumesic DA. Assessment of cardiovascular risk and prevention of cardiovascular disease in women with the polycystic ovary syndrome: a consensus statement by the Androgen Excess and Polycystic Ovary Syndrome (AE-PCOS) Society. J Clin Endocrinol Metab. 2010 May;95(5):2038-49. doi: 10.1210/jc.2009-2724. Epub 2010 Apr 7.

76: Oh JY, Lee JA, Lee H, Oh JY, Sung YA, Chung H. Serum C-reactive protein levels in normal-weight polycystic ovary syndrome. Korean J Intern Med. 2009 Dec;24(4):350-5. doi: 10.3904/kjim.2009.24.4.350. Epub 2009 Nov 27.

77: Rachoń D, Teede H. Ovarian function and obesity--interrelationship, impact on women's reproductive lifespan and treatment options. Mol Cell Endocrinol. 2010 Mar 25;316(2):172-9. doi: 10.1016/j.mce.2009.09.026. Epub 2009 Oct 7.

78: Erdogan M, Karadeniz M, Berdeli A, Tamsel S, Yilmaz C. The relationship of the interleukin-6 -174 G>C gene polymorphism with cardiovascular risk factors in Turkish polycystic ovary syndrome patients. Int J Immunogenet. 2009 Oct;36(5):283-8. doi: 10.1111/j.1744-313X.2009.00867.x.

79: Cetinkalp S, Erdogan M, Karadeniz M, Berdeli A, Tamsel S, Ozgen AG, Saygili F, Yilmaz C. The relationship of the Fas 670 A/G gene polymorphism with cardiovascular risk factors in polycystic ovary syndrome (PCOS) patients. Gynecol Endocrinol. 2010 Mar;26(3):167-72. doi: 10.1080/09513590903215508.

80: Akram T, Hasan S, Imran M, Karim A, Arslan M. Association of polycystic ovary syndrome with cardiovascular risk factors. Gynecol Endocrinol. 2010 Jan;26(1):47-53. doi: 10.3109/09513590903159565.

81: Güven A, Ozgen T, Aliyazicioğlu Y. Adiponectin and resistin concentrations after glucose load in adolescents with polycystic ovary syndrome. Gynecol Endocrinol. 2010 Jan;26(1):30-8. doi: 10.3109/09513590903159540.

82: Shi D, Dyck MK, Uwiera RR, Russell JC, Proctor SD, Vine DF. A unique rodent model of cardiometabolic risk associated with the metabolic syndrome and polycystic ovary syndrome. Endocrinology. 2009 Sep;150(9):4425-36. doi:

10.1210/en.2008-1612. Epub 2009 May 21.

83: Cahill D. PCOS. BMJ Clin Evid. 2009 Jan 15;2009. pii: 1408.

84: Smiley DA, Khalil RA. Estrogenic compounds, estrogen receptors and vascular cell signaling in the aging blood vessels. Curr Med Chem. 2009;16(15):1863-87.

85: Thomson RL, Buckley JD, Moran LJ, Noakes M, Clifton PM, Norman RJ, Brinkworth GD. Comparison of aerobic exercise capacity and muscle strength in overweight women with and without polycystic ovary syndrome. BJOG. 2009 Aug;116(9):1242-50. doi: 10.1111/j.1471-0528.2009.02177.x. Epub 2009 May 11.

86: Moran L, Teede H. Metabolic features of the reproductive phenotypes of polycystic ovary syndrome. Hum Reprod Update. 2009 Jul-Aug;15(4):477-88. doi: 10.1093/humupd/dmp008. Epub 2009 Mar 11.

87: Kassi E, Diamanti-Kandarakis E. The effects of insulin sensitizers on the cardiovascular risk factors in women with polycystic ovary syndrome. J Endocrinol Invest. 2008 Dec;31(12):1124-31.

88: Moini A, Eslami B. Familial associations between polycystic ovarian syndrome and common diseases. J Assist Reprod Genet. 2009 Mar;26(2-3):123-7. doi: 10.1007/s10815-009-9297-7. Epub 2009 Feb 10.

89: Soares GM, Vieira CS, Martins WP, Franceschini SA, dos Reis RM, Silva de Sá MF, Ferriani RA. Increased arterial stiffness in nonobese women with polycystic ovary syndrome (PCOS) without comorbidities: one more characteristic inherent to the syndrome? Clin Endocrinol (Oxf). 2009 Sep;71(3):406-11. doi: 10.1111/j.1365-2265.2008.03506.x. Epub 2008 Dec 15.

90: Moran LJ, Hutchison SK, Meyer C, Zoungas S, Teede HJ. A comprehensive assessment of endothelial function in overweight women with and without polycystic ovary syndrome. Clin Sci (Lond). 2009 May;116(10):761-70. doi: 10.1042/CS20080218.

91: Westerveld HE, Hoogendoorn M, de Jong AW, Goverde AJ, Fauser BC, Dallinga-Thie GM. Cardiometabolic abnormalities in the polycystic ovary syndrome: pharmacotherapeutic insights. Pharmacol Ther. 2008 Sep;119(3):223-41. doi: 10.1016/j.pharmthera.2008.04.009. Epub 2008 Jun 17.

92: Erdoğan M, Karadeniz M, Alper GE, Tamsel S, Uluer H, Cağlayan O, Saygili F, Yilmaz C. Thrombin-activatable fibrinolysis inhibitor and cardiovascular risk factors in polycystic ovary syndrome. Exp Clin Endocrinol Diabetes. 2008 Mar;116(3):143-7. doi: 10.1055/s-2007-992118.

93: Andiran N, Yordam N. Lipoprotein(a) levels in girls with premature adrenarche. J Paediatr Child Health. 2008 Mar;44(3):138-42. doi: 10.1111/j.1440-1754.2007.01210.x.

94: Palep-Singh M, Picton HM, Yates ZR, Barth JH, Balen AH. Plasma homocysteine concentrations and the single nucleotide polymorphisms in the methionine synthase gene (MTR 2756A>G): Associations with the polycystic ovary syndrome An observational study. Eur J Obstet Gynecol Reprod Biol. 2008 Jun;138(2):180-6. doi: 10.1016/j.ejogrb.2007.12.015. Epub 2008 Feb 20.

95: García-Romero G, Escobar-Morreale HF. Hyperandrogenism, insulin resistance and hyperinsulinemia as cardiovascular risk factors in diabetes mellitus. Curr Diabetes Rev. 2006 Feb;2(1):39-49.

96: Cascella T, Palomba S, De Sio I, Manguso F, Giallauria F, De Simone B, Tafuri D, Lombardi G, Colao A, Orio F. Visceral fat is associated with cardiovascular risk in women with polycystic ovary syndrome. Hum Reprod. 2008 Jan;23(1):153-9. Epub 2007 Nov 16.

97: Hunter A, Vimplis S, Sharma A, Eid N, Atiomo W. To determine whether first-degree male relatives of women with polycystic ovary syndrome are at higher risk of developing cardiovascular disease and type II diabetes mellitus. J Obstet Gynaecol. 2007 Aug;27(6):591-6.

98: Lunde O, Tanbo T. Polycystic ovary syndrome: a follow-up study on diabetes mellitus, cardiovascular disease and malignancy 15-25 years after ovarian wedge resection. Gynecol Endocrinol. 2007 Dec;23(12):704-9.

99: Krentz AJ, von Mühlen D, Barrett-Connor E. Searching for polycystic ovary syndrome in postmenopausal women: evidence of a dose-effect association with prevalent cardiovascular disease. Menopause. 2007 Mar-Apr;14(2):284-92.

100: Brinkworth GD, Noakes M, Moran LJ, Norman R, Clifton PM. Flow-mediated dilatation in overweight and obese women with polycystic ovary syndrome. BJOG. 2006 Nov;113(11):1308-14.

101: Sorensen MB, Franks S, Robertson C, Pennell DJ, Collins P. Severe endothelial dysfunction in young women with polycystic ovary syndrome is only

partially explained by known cardiovascular risk factors. Clin Endocrinol (Oxf). 2006 Nov;65(5):655-9.

102: ESHRE Capri Workshop Group. Hormones and cardiovascular health in women. Hum Reprod Update. 2006 Sep-Oct;12(5):483-97. Epub 2006 Jun 28.

103: Tan S, Hahn S, Janssen OE. Insulin resistance syndrome and polycystic ovary syndrome: implications for diagnosis and treatment. Panminerva Med. 2005 Dec;47(4):211-7.

104: Kacalska O, Krzyczkowska-Sendrakowska M, Milewicz T, Zabińska-Popiela M, Bereza T, Krzysiek-Maczka G, Krzysiek J. [Molecular action of insulin-sensitizing agents]. Endokrynol Pol. 2005 May-Jun;56(3):308-13.

105: Creatsas G, Christodoulakos G, Lambrinoudaki I. Cardiovascular disease: screening and management of the a-symptomatic high-risk post-menopausal woman. Maturitas. 2005 Nov 15;52 Suppl 1:S32-7. Epub 2005 Sep 2.

106: Marsh K, Brand-Miller J. The optimal diet for women with polycystic ovary syndrome? Br J Nutr. 2005 Aug;94(2):154-65.

107: Meyer C, McGrath BP, Teede HJ. Overweight women with polycystic ovary syndrome have evidence of subclinical cardiovascular disease. J Clin Endocrinol Metab. 2005 Oct;90(10):5711-6. Epub 2005 Jul 26.

108: Margolin E, Zhornitzki T, Kopernik G, Kogan S, Schattner A, Knobler H. Polycystic ovary syndrome in post-menopausal women--marker of the metabolic syndrome. Maturitas. 2005 Apr 11;50(4):331-6.

109: Weerakiet S. Polycystic ovary syndrome and the metabolic syndrome. J Med Assoc Thai. 2004 Oct;87 Suppl 3:S189-93.

110: Boulman N, Levy Y, Leiba R, Shachar S, Linn R, Zinder O, Blumenfeld Z. Increased C-reactive protein levels in the polycystic ovary syndrome: a marker of cardiovascular disease. J Clin Endocrinol Metab. 2004 May;89(5):2160-5.

111: Taponen S, Martikainen H, Järvelin MR, Sovio U, Laitinen J, Pouta A, Hartikainen AL, McCarthy MI, Franks S, Paldanius M, Ruokonen A; Northern Finland Birth Cohort 1966 Study. Metabolic cardiovascular disease risk factors in women with self-reported symptoms of oligomenorrhea and/or hirsutism: Northern Finland

Birth Cohort 1966 Study. J Clin Endocrinol Metab. 2004 May;89(5):2114-8.

112: Pelikánová T. [The metabolic syndrome]. Vnitr Lek. 2003 Dec;49(12):900-6. Review. Czech.

113: Legro RS. Polycystic ovary syndrome and cardiovascular disease: a premature association? Endocr Rev. 2003 Jun;24(3):302-12. Review.

114: Hu FB. Overweight and obesity in women: health risks and consequences. J Womens Health (Larchmt). 2003 Mar;12(2):163-72.

115: Yildiz BO, Haznedaroğlu IC, Kirazli S, Bayraktar M. Global fibrinolytic capacity is decreased in polycystic ovary syndrome, suggesting a prothrombotic state. J Clin Endocrinol Metab. 2002 Aug;87(8):3871-5.

116: Cibula D, Cífková R, Fanta M, Poledne R, Zivny J, Skibová J. Increased risk of non-insulin dependent diabetes mellitus, arterial hypertension and coronary artery disease in perimenopausal women with a history of the polycystic ovary syndrome. Hum Reprod. 2000 Apr;15(4):785-9.

117: Solomon CG, Hu FB, Dunaif A, Rich-Edwards JE, Stampfer MJ, Willett WC, Speizer FE, Manson JE. Menstrual cycle irregularity and risk for future cardiovascular disease. J Clin Endocrinol Metab. 2002 May;87(5):2013-7.

118: Wild S, Pierpoint T, McKeigue P, Jacobs H. Cardiovascular disease in women with polycystic ovary syndrome at long-term follow-up: a retrospective cohort study. Clin Endocrinol (Oxf). 2000 May;52(5):595-600.

119: Birdsall MA, Farquhar CM, White HD. Association between polycystic ovaries and extent of coronary artery disease in women having cardiac catheterization. Ann Intern Med. 1997 Jan 1;126(1):32-5.

120: Dahlgren E, Janson PO, Johansson S, Lapidus L, Lindstedt G, Tengborn L. Hemostatic and metabolic variables in women with polycystic ovary syndrome. Fertil Steril. 1994 Mar;61(3):455-60.
